# Electro-Persulfate Processes for the Treatment of Complex Wastewater Matrices: Present and Future

**DOI:** 10.3390/molecules26164821

**Published:** 2021-08-09

**Authors:** Annabel Fernandes, Maria João Nunes, Ana Sofia Rodrigues, Maria José Pacheco, Lurdes Ciríaco, Ana Lopes

**Affiliations:** FibEnTech-UBI, Department of Chemistry, Universidade da Beira Interior, 6201-001 Covilhã, Portugal; annabelf@ubi.pt (A.F.); maria.nunes@ubi.pt (M.J.N.); ana.sf.rodrigues@ubi.pt (A.S.R.); mjap@ubi.pt (M.J.P.); lciriaco@ubi.pt (L.C.)

**Keywords:** sulfate radical-based processes, electrochemical persulfate activation, hybrid persulfate activation, recalcitrant wastewaters

## Abstract

Complex wastewater matrices present a major environmental concern. Besides the biodegradable organics, they may contain a great variety of toxic chemicals, heavy metals, and other xenobiotics. The electrochemically activated persulfate process, an efficient way to generate sulfate radicals, has been widely applied to the degradation of such complex effluents with very good results. This review presents the fundamentals of the electro-persulfate processes, highlighting the advantages and limitations, followed by an exhaustive evaluation on the application of this process for the treatment of complex industrial effluents. An overview of the main relevant experimental parameters/details and their influence on the organic load removal is presented and discussed, having in mind the application of these technologies at an industrial scale. Finally, the future perspectives for the application of the electro-persulfate processes in the treatment of complex wastewater matrices is outlined.

## 1. Introduction

Increasing industrialization and urbanization results in the generation of large volumes of wastewaters, which represent a major environmental concern, due to their possible release in the environment without the appropriate treatment. Depending on its origin, municipal, livestock, refinery, industrial or food-processing activities, among others, wastewaters can present, in its composition, biodegradable organics, a great variety of toxic chemicals, heavy metals, and other xenobiotics [1]. Most of these compounds are persistent and adversely affect human health and aquatic biota, imparting genotoxicity, endocrine disruption, and bioaccumulation, which makes it imperative to treat the wastewaters before their discharge into water courses [1]. 

Conventional wastewater treatments often lack any capability to treat complex wastewaters that contain a mixture of refractory and non-biodegradable compounds. Thus, in the past years, several studies have been focused on the development of new technologies that enable the efficient treatment of these complex wastewater matrices to comply with the discharge regulations. Among the studied technologies, advanced oxidation processes (AOPs) have gained increasing attention, due to their good performance in the removal of recalcitrant pollutants. Traditionally, AOPs are based on the in situ generation of the non-selective strong oxidant hydroxyl radical (HO^•^), with a redox potential of 1.8–2.7 V, which unselectively promotes the partial or complete mineralization of a wide range of organic compounds [2]. However, more recently, sulfate radical-based AOPs have emerged, as a sub-category of the AOPs, where the generation of the sulfate radical (SO_4_^•−^) is promoted, alone or combined with hydroxyl radicals [3,4]. 

Among the different SO_4_^•−^ generation processes, the electrochemical ones, usually designated as electro-persulfate processes, were extensively investigated and considered the most efficient for the formation of sulfate radicals [5]. There are several studies reporting the application of electro-persulfate processes in the treatment of wastewaters with recalcitrant properties, with very promising results, making this technology a possible solution for the remediation of complex wastewater matrices.

The aim of this paper is to present a general review of the most relevant applications of the electro-persulfate processes in the treatment of complex wastewater matrices. To better understand their advantages and limitations for wastewater treatment, the fundamentals are briefly presented. The studies found in the literature revision for the application of such technologies to the treatment of complex wastewaters are discussed, to point out the most relevant conclusions. Finally, the future perspectives for application of the electro-persulfate processes at an industrial scale are presented.

## 2. Fundamentals of the Electro-Persulfate Processes

Electro-persulfate processes consist in the electrochemical activation of persulfate (peroxymonosulfate-PMS, HSO_5_^−^, E° = 1.82 V; or peroxydisulfate—PDS, S_2_O_8_^2−^, E° = 2.01 V) to generate the highly reactive sulfate radical, which presents a redox potential of 2.5–3.1 V) [4]. Both PMS and PDS contain a peroxide bond (−O−O−) that can be electrochemically cleaved by the reaction with an electron to form SO_4_^•−^ (Equations (1) and (2), respectively) [5,6]. For this purpose, persulfate (PS) can be externally added to the system or it can be electrochemically produced, in sulfate-containing solutions, from the oxidation of SO_4_^2−^ and HSO_4_^−^ ions, through the reactions described by the Equations (3) and (4), respectively [7].

HSO_5_^−^ + e^−^ → SO_4_^•−^ + HO^−^, (1)

S_2_O_8_^2−^ + e^−^ → SO_4_^•−^ + SO_4_^2^^−^, (2)

2SO_4_^2^^−^ → S_2_O_8_^2−^ + 2e^−^, (3)

2HSO_4_^−^ → S_2_O_8_^2−^ + 2H^+^ + 2e^−^. (4)

When wastewater is rich in sulfate, in situ persulfate electrogeneration benefits from saving chemicals and the associated cost. Moreover, when utilizing anode materials with high oxygen evolution potential (OEP), such as platinum (Pt), boron-doped diamond (BDD), PbO_2_, SnO_2_, and Ti_4_O_7_, additional sulfate radical electrogeneration can be accomplished via sulfate ions direct oxidation (Equation (5)) and by oxidation through a hydroxyl radical (Equation (6)) [8,9]. Supplementary persulfate ions can be also obtained through sulfate ions oxidation by hydroxyl radical (Equation (7)) [8].

SO_4_^2−^ → SO_4_^•−^ + e^−^, (5)

SO_4_^2^^−^ + OH^•^ → SO_4_^•−^ + OH^−^, (6)

2SO_4_^2^^−^ + 2OH^•^ → S_2_O_8_^2^^−^ + 2OH^−^. (7)

In the electro-persulfate process with external PS addition, the most utilized PMS or PDS salts are: oxone salt (KHSO_5_·0.5KHSO_4_·0.5K_2_SO_4_); potassium peroxydisulfate (K_2_S_2_O_8_); sodium peroxydisulfate (Na_2_S_2_O_8_); and ammonium peroxydisulfate ((NH_4_)_2_S_2_O_8_). PDS salts are more stable, environmentally friendly, and cheap compared to PMS salts, being preferably used in sulfate radical-based advanced oxidation processes [7]. Among the PDS salts, sodium peroxydisulfate is pointed as the most suitable for water and wastewater treatment, since it presents higher solubility in water than potassium peroxydisulfate, and ammonium peroxydisulfate has the disadvantage of reacting with the ammonium ions, thus reducing PDS availability to form sulfate radicals [7].

Electrochemically produced or externally added, when activated, PS originates the strong and highly oxidizing sulfate radical (Equations (1) and (2)), which presents higher redox potential than that of a hydroxyl radical, promoting the nonselective oxidation and efficient removal of a wide range of organic compounds [4,5]. Moreover, the half-life time of SO_4_^•−^ is usually referred as being longer than that of HO^•^, enabling better mass transfer performance and contact with the organic compounds in solution, promoting their complete or partial mineralization [5]. This high efficiency in pollutants removal is further enhanced by secondary radicals, generated from a sulfate radical reaction with water, for instance, to give hydroxyl radical (Equation (8)). In fact, SO_4_^•−^ readily reacts with water at a wide range of pH values [5]. According to the literature, hydroxyl radicals are the primary reactive species under alkaline pH conditions [10]. At neutral pH values, hydroxyl and sulfate radicals participate equally in reactions, with sulfate radicals being the dominant reactive species at pH < 7 [10]. If high-OEP anode materials are utilized in persulfate activated processes, hydroxyl radicals, formed at the anode surface (Equation (9)), will also contribute to the degradation of the organic matter, additionally to the oxidation by sulfate radicals or by direct electron transfer.

SO_4_^•−^ + H_2_O → SO_4_^2^^−^ + HO^•^ + H^+^, (8)

H_2_O → HO^•^ + H^+^ + e^−^. (9)

**Figure 1:**
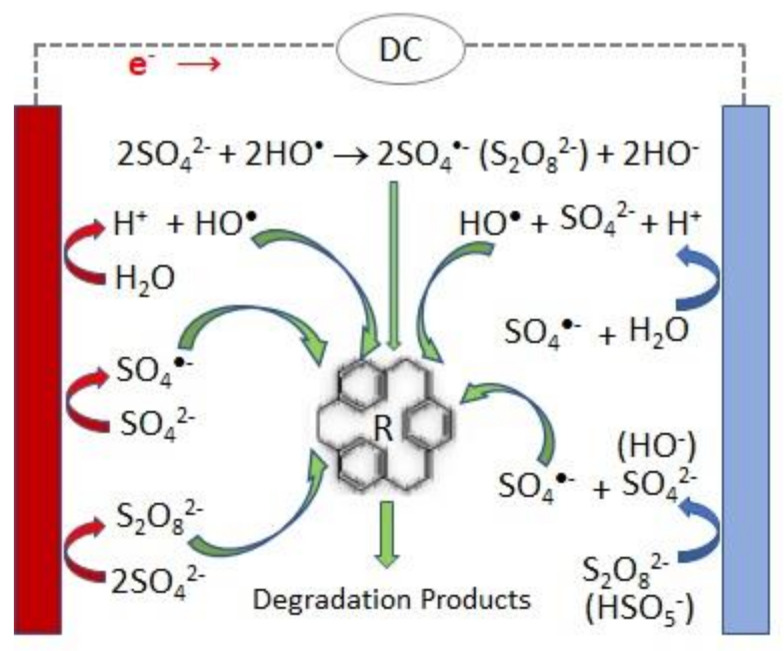
Scheme of the main reactions involved in the persulfate electrochemical production and activation.

A scheme of the main reactions involved in the persulfate electrochemical production and activation is presented in Figure 1.

Attending that persulfate can be electrochemically generated from sulfate ions (Equation (3)), it has been suggested that PS can be regenerated at the anode after completion of the sulfate radical reactions with water, organics, etc., thus enabling a perpetual source of sulfate radicals [9,10].

Persulfate electrochemical activation efficiency strongly depends on the electrode material, among other factors. BDD has been described in the literature as one of the most efficient and inert anode materials for the electrochemical sulfate radical-based processes. Nonetheless, mass transfer limitations have been reported, due to the fact that electrochemical activation reactions mainly occur at the electrode’s surface, hindering high current efficiencies of the electro-activated PS reactions and, consequently, leading to long treatment times and energy consumptions [5].

The combination of electrochemical activation with other PS activation methods has been widely studied with the aim of enhance the PS activation efficiency. Besides electrochemical activation, PS can be activated by transition metals, non-metal catalysts, UV or visible radiation, ultrasound (US), microwave, alkaline medium, and heat. Each one of these methods has advantages and shortcomings for the PS activation [4]. So, the combination of different activation methods can make full use of their advantages and overcome the drawbacks.

Persulfate can be activated through one-electron transfer using metals (M) such as silver, copper, iron, zinc, cobalt, and manganese to form the sulfate radical (Equations (10) and (11)) [10].

HSO_5_^−^ + M^n^ → SO_4_^•−^ + OH^−^ + M^n+1^, (10)

S_2_O_8_^2^^−^ + M^n^ → SO_4_^•−^ + SO_4_^2^^−^ + M^n+1^. (11)

**Figure 2:**
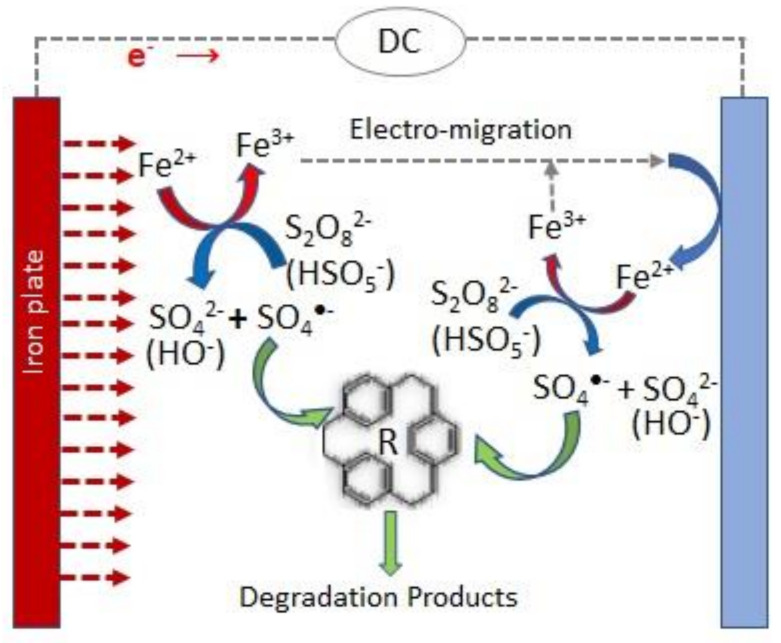
Scheme of the main reactions involved in the persulfate electrochemical activation when sacrificial iron anodes are used.

One of the most studied hybrid processes to activate persulfate, involving transition metal and electrochemical activation, comprises the use of sacrificial transition metal anodes. Iron and aluminum are the most commonly applied anode materials, with Fe being the most preferred due to its effectiveness in PS activation, its relatively non-toxic and environmentally friendly nature, and its lower cost compared to other transition metals [11,12]. Figure 2 presents a scheme of an electrochemical cell that uses a sacrificial iron anode and the main reactions occurring in this combined PS activation process.

When sacrificial iron anodes are utilized, Fe^2+^ is electrochemically produced through anodic dissolution (Equation (12)) and activates persulfate ions through the reactions described in Equations (13) and (14) [12,13].

Fe^0^ → Fe^2+^ + e^−^, (12)

HSO_5_^−^ + Fe^2+^ → SO_4_^•−^ + OH^−^ + Fe^3+^, (13)

S_2_O_8_^2^^−^ + Fe^2+^ → SO_4_^•−^ + SO_4_^2^^−^ + Fe^3+^. (14)

According to the Faraday’s law, iron anodic dissolution rate is proportional to the applied current intensity. Thus, the electrolytic supply of Fe^2+^ can be easily controlled by adjusting this operational parameter, which optimizes the utilization of both Fe^2+^ and PS [14]. Furthermore, in the electrochemical cell, Fe^2+^ can be regenerated through the Fe^3+^ reduction at the cathode, overcoming the problem raised by the slow Fe^2+^ regeneration in the conventional PS activation through ferrous ion addition, enhancing the PS activation and, consequently, the sulfate radical generation [11,12,13]. In this electrochemical process, iron hydroxides are also formed, leading to electrocoagulation and electroflotation, alongside electrochemical oxidation [13,15]. 

Besides the use of sacrificial metal anodes, PS activation by combined transition metal and electrochemical process can be accomplished by adding transition-metal salts, mineral metal-based activators, or other metal oxides and heterogeneous metal-based catalysts to the solution [4,10]. Heterogeneous metal PS activation has been regarded as more advantageous than homogeneous since it avoids the formation of metal hydroxide sludge and enables the recycling of the heterogeneous catalysts [16]. Persulfate activation through iron-containing heterogeneous catalysts can be generally described by Equation (15) to (20) [17,18,19].

Fe(0) + HSO_5_^−^ + 2H^+^ → Fe(II) + HSO_4_^−^ + H_2_O, (15)

Fe(0) + 2S_2_O_8_^2^^−^ → Fe(II) + 2SO_4_^•−^ + 2SO_4_^2^^−^, (16)

Fe(II) + HSO_5_^−^ → Fe(III) + SO_4_^•−^ + OH^−^, (17)

Fe(II) + S_2_O_8_^2^^−^ → Fe(III) + SO_4_^•−^ + SO_4_^2^^−^, (18)

Fe(III) + HSO_5_^−^ → Fe(II) + SO_5_^•−^ + H^+^, (19)

Fe(III) + S_2_O_8_^2^^−^ → Fe(II) + S_2_O_8_^•−^. (20)

Another hybrid PS electro-activation process that has been widely studied for the treatment of complex wastewater matrices utilizes UV or US irradiation. UV irradiation is regarded as a benign and cost-effective method to activate persulfate, although it exhibits low removal efficacies for some organic contaminants [4]. In this activation process, two mechanisms might be involved: the cleavage of the peroxide bond by energy input from the UV irradiation (Equations (21) and (22)); and electron transfer through the electron produced from water exposure to UV (Equations (23) to (25)) [4]. Persulfate activation through UV irradiation is typically implemented at the wavelength of 254 nm, due to the diapason high energy and SO_4_^•−^ absorptivity and maximum quantum [4].

HSO_5_^−^ + energy → SO_4_^•−^ + HO^•^, (21)

S_2_O_8_^2^^−^ + energy → 2SO_4_^•−^, (22)

H_2_O + UV → H^•^ + HO^•^, (23)

HSO_5_^−^ + H^•^ → SO_4_^•−^ + H_2_O, (24)

S_2_O_8_^2^^−^ + H^•^ → SO_4_^•−^ + SO_4_^2^^−^ + H^+^. (25)

Besides its potential in PS activation, UV irradiation has also been employed in hybrid PS electro-activation processes to enhance the catalytic activity of heterogeneous metal-based persulfate activators [20]. The utilization of UV radiation to activate PS may present a disadvantage for dark wastewaters or effluents containing suspended solids, because radiation may be absorbed without activating PS. 

The PS activation through US irradiation (Equations (21) and (22)), often referred to as a type of heat activation, is a result of extreme temperature and pressure rises, due to cavitation [21,22]. One of the main advantages of coupling ultrasound with electrochemical PS activation, besides the additional persulfate activation, is that ultrasonic irradiation accelerates the mass transfer rate of persulfate anions in solution towards the cathode, by acoustic streams [22].

Though less applied in hybrid persulfate electro-activation processes, traditional heat is one of the simplest methods for PS activation [23]. The energy input by the high temperature (>50 °C) causes the cleavage of the persulfate peroxide bond and sulfate radicals are formed according to Equations (21) and (22) [4]. Although heat is effective in PS activation, the energy demand is too high, which makes this method unfeasible for remediation processes on a large scale [4]. The introduction of an electrochemically assisted persulfate activation enhances the sulfate radical production and reduces the energy consumption of the heat-activated process [23].

Section 3 presents the main studies performed on the treatment of complex wastewater matrices by electro-persulfate processes, as well as the most relevant results and conclusions obtained.

## 3. Treatment of Complex Wastewater Matrices by Electro-Persulfate Processes

### 3.1. Single Electrolytic Activation of Persulfate

Electrolytic activation of persulfate has been successfully applied in the treatment of complex wastewater matrices, such as dyeing wastewater [24], industrial effluent containing dinitrotoluenes [25], washing machine effluent [26], oil sands process water [8], and cyanide-containing wastewater [27]. The conditions used in the experiments and the main results obtained are summarized in Table 1. All the studies presented were conducted at laboratory scale, in batch mode conditions. The anode material employed was Pt or BDD and, in most studies, the persulfate source was PDS. Added PS concentration, applied current, temperature, and initial pH conditions were the main variables studied.

Chanikya and collaborators [24] studied the treatment of a dyeing effluent by electro-persulfate degradation and evaluated the influence of the added PDS concentration (0, 0.5 and 1 g L^−1^). The authors observed that the maximum chemical oxygen demand (COD) removal (76% in a 1-h assay) was attained with 0.5 g L^−1^ of PDS, although, during the first part of the assay, COD removal rate was higher for 1 g L^−1^. These results were explained by the occurrence of the side reactions presented in Equations (26) and (27), which are favored at elevated persulfate concentrations, reducing sulfate radical concentration. A COD removal of 56%, in a 1-h assay, was observed in the experiments without added PDS, i.e., the pollutant load removal ascribed to anodic oxidation.

SO_4_^•−^ + S_2_O_8_^2^^−^ → SO_4_^2^^−^ + S_2_O_8_^•−^, (26)

SO_4_^•−^ + SO_4_^•−^ → S_2_O_8_^2^^−^. (27)

The influence of applied potential (E), temperature, initial pH, and PDS concentration, in the electro-persulfate degradation of an effluent containing dinitrotoluenes (DNTs), obtained from a military ammunition plant, was assessed by Chen et al. [25]. When compared with standalone electrolysis or persulfate oxidation, under identical experimental conditions (T = 303 K; pH = 0.5), electro-activated persulfate experiments presented much higher total organic carbon (TOC) abatement: 45% for electrolysis at 6 V; 4% with 1.0 wt% PDS; 70% for electro-activated persulfate at 6 V with 1.0 wt% PDS. The increase in TOC removal for the electro-activated persulfate process was ascribed to the stronger oxidation power of sulfate radicals formed by reduction in persulfate anions. The best TOC removal, 95%, was attained for the lowest pH tested and the highest added PDS concentration, applied potential, and temperature.

The formation of hydrogen peroxide by the cathodic reduction in dissolved oxygen (Equation (28)), from water oxidation at the anode (Equation (29)), was analyzed, and the results were discussed based on the possibility that DNTs were, simultaneously, oxidized by H_2_O_2_. According to the authors, this cathodic reaction (Equation (28)) may compete with radical sulfate formation.

O_2_ + 2H^+^ + 2e^−^ → H_2_O_2_, (28)

2H_2_O → O_2_ + 4H^+^ + 4e^−^. (29)

The authors also studied the influence of bubbling oxygen or nitrogen in the reactor by providing oxygen/nitrogen at flow rates of 50–150 mL min^−1^, and have found that bubbling gaseous N_2_ can significantly improve sulfate radical formation rate by depleting dissolved O_2_ concentration, thus reducing H_2_O_2_ formation [25].

In a different study, utilizing an organic wastewater with high concentration in cyanide (CNW), Yang and collaborators [27] also evaluated the influence of applied current, temperature, and initial pH in the single electrolytic activation of PDS, and compared the results with electrooxidation (EO) and PDS oxidation stand-alone. After 24 h assays, the COD removals were: PDS (0.1 M)—36.9%, EO (20 mA cm^−2^)—82.3 %, EO + PDS (20 mA cm^−2^, 0.1 M)—99.5%. Although EO + PDS was the most effective process in COD removal, the synergistic effect was not observed, and the authors attribute this result to the side reactions that take place in the different processes. However, the synergistic effect was observed in the electrical energy consumption: EO—245.4 kWh m^−3^ order^−1^; EO + PDS—62.5 kWh m^−3^ order^−1^.

Utilizing a different persulfate source, Ghanbari and Martínez-Huitle [26] studied the treatment of a washing machine effluent by electro-persulfate degradation through PMS addition. In the absence of PMS, only 20% COD abatement was attained after 3-h assays. The COD removal was enhanced by the PMS addition and, for similar experimental conditions, a value of 32.7% was obtained. According to the authors, without PMS, the formed hydroxyl radicals were chemisorbed on an Pt anode, meaning its oxidation ability was very limited, although active chlorine species may have been formed due to the initial chloride concentration (243 mg L^−1^). When PMS is added, it can be activated by reduction at the cathode to form sulfate radicals, enhancing the COD removal rate. Additionally, PMS may react with chloride ions to form HOCl (Equation (30)), himself a powerful oxidant.

HSO_5_^−^ + Cl^−^ → HOCl+ SO_4_^2^^−^. (30)

The generation of persulfate ions and sulfate radicals from the electrochemical oxidation of a sulfate-containing wastewater, and their involvement on the pollutants degradation mechanism, has also been the focus of a study [8]. The depuration of oil sands process water (OSPW) was performed by electrooxidation, and electron paramagnetic resonance spectrometry and spectrophotometry were utilized to study the involvement in the degradation mechanism of radicals (HO^•^, SO_4_^•−^, CO_3_^•−^ electrogenerated at BDD from H_2_O, SO_4_^2^^−^ and CO_3_^2^^−^, respectively) and non-radicals (S_2_O_8_^2−^, C_2_O_6_^2−^, and active chlorine electrogenerated at BDD from SO_4_^2−^, CO_3_^2−^ and Cl^−^, respectively) species, by utilizing different radical scavengers [8]. OSPW contained suspended solids (62.5 mg L^−1^), salts (chloride-900, sulfate-650, carbonate-1500, nitrate-18 mg L^−1^), trace metals, inorganic compounds, and organic compounds (naphthenic acids (NAs), polycyclic aromatic hydrocarbons (PAHs) and estrogenic compounds). Although PS was not added to the samples in this study, it could be formed from the oxidation of SO_4_^2−^ (Equations (3) and (7)) and in the presence of enough amounts of SO_4_^•−^ (Equation (27)). In fact, persulfate was detected in the study and, at a 0.5-h assay, its concentration was 1.5 mM for 20 and 30 mA cm^−2^, and 0.25 mM for 10 mA cm^−2^ at 1 h. Persulfate generation enhancement at a higher applied current density was attributed to the increase in reaction rate of Equations (3), (7), and (30). Although other species than sulfate radical were involved in the degradation of the OSPW, NAs, and PAHs were completely degraded after a 2-h assay at a current density of 5 mA cm^−2^ or higher, and 100% TOC removal was obtained for a 6-h assay.

Thus, in the processes with single electrolytic persulfate activation, applied current has a positive effect on the organic load removal. When Pt is the anode material, this happens probably because higher potential difference induces higher formation rate of sulfate radicals, since it is assumed that degradation by anodic oxidation at Pt is negligible [25]. If anode materials, such as BDD, are utilized, COD and TOC removal increase with applied current density due to direct and indirect oxidation of the pollutants. However, degradation rate is gradually reduced during the assay, due to mass transport limitations when the organic load became lower [27].

The increase in PS concentration may have a positive effect on the organic load removal in processes with single electrolytic persulfate activation. In fact, Chen and collaborators [25] observed an increase in TOC abatement when PDS concentration varied from 0.7 to 1.7 wt%, probably due to the increase in sulfate radical formation rate. However, authors refer that an increase in PDS concentration to 2.1 wt% decreases the degradation rate, due to the side reaction between the excess in persulfate anions and sulfate radicals (Equation (26)).

Temperature also has a positive effect on the organic load abatement, due to the increase in the kinetic constant of the degradation reaction by a sulfate radical. Moreover, oxygen solubility decreases with temperature, leading to lower electrogenerated H_2_O_2_, which points to a degradation mechanism mainly ascribed to the sulfate radical as temperature increases [25]. However, for high increases in temperature, only small increases in organic load removal may happen, probably because higher temperatures promote radicals quenching reactions [27].

Regarding the influence of pH, degradation rate decreases with pH increases; this decrease is ascribed to the lower hydrogen peroxide formation at higher pH values [25]. In the presence of carbonates, organic degradation rate is also favored at acid conditions, since carbonate and bicarbonate concentration decrease, thus contributing less to the inhibition of persulfate and hydroxyl radicals. Energy consumption also increases with pH [27].

Concerning energy consumption in processes with single electrolytic persulfate activation, Chanikya et al. [24], in the treatment of a dyeing effluent, observed that PDS addition enhanced instantaneous current efficiency (ICE), which increased with time, mainly at PDS concentration of 1 g L^−1^, showing the effective oxidation by the sulfate radicals that are generated near the cathode, by persulfate reduction. When the oxidation was run without persulfate addition, i.e., in anodic oxidation, ICE increased during the first 30 min, and then decreased, due to mass transport limitation, since the main oxidant, hydroxyl radical, has a short lifespan and acts mainly in the vicinity of the electrode’s surface. However, according to the authors, active chlorine species, formed due to the high chloride concentration in the effluent (1845 mg L^−1^), also participated in the oxidation pathway.

When analyzing the processes just described, one must have in mind that this reduction in energy consumption results from the electrical potential reduction when persulfate is added, due to a conductivity increase. So, the final pH of the treated effluent, which may need correction, and the persulfate price must be variables to consider when costs are estimated. Lower energy consumption may also be attained at higher temperatures, if the energy spent in heating the wastewater is disregarded [27]. On the other hand, energy consumption increases with current density, due to the potential difference increase and side reactions [27].

### 3.2. Combined Electrolytic and Metal Activation of Persulfate

#### 3.2.1. Persulfate Electro-Activation through Sacrificial Anodes

Persulfate activation through metal sacrificial anodes has been applied in the treatment of different types of effluents, with COD values between 0.5 and 128 g L^−1^, namely: sanitary landfill leachate (SLL) [28], nanofiltration concentrate from SLL [11,29,30], biodiesel wastewater [31,32], pulp and paper industry effluents [12,15], grey water [13], palm oil industry effluent [33], and car wash wastewater [34]. The main data from these studies are presented in Table 2.

Different reactor designs were utilized in the treatment of these effluents, the most common set-up two parallel electrodes. Nonetheless, electrochemical cells containing four monopolar parallel electrodes were also utilized [11,15,29,30]. 

In the different studies, the most utilized metal, as a consumable anode material, was iron, followed by aluminum, and, although the majority of these works have used identical material for anode and cathode, other cathode materials, such as graphite, copper, or metallic oxides, were also reported. Varank et al. [15] used iron and aluminum in a comparative study for the treatment of a paper industry wastewater. The best COD removals attained with iron and aluminum consumable anodes were 51 and 81%, respectively, making the use of aluminum electrodes more advantageous. According to the authors, since electrocoagulation occurs simultaneously with persulfate activation by metal ions, the best performance obtained with the aluminum consumable anodes may be ascribed to the electrocoagulation process with aluminum when compared to iron. 

The inter-electrode gap ranges between 1 and 4 cm for the different studies presented. The influence of this operational parameter in the electro-persulfate activation process was only assessed by Durna and Genç (data not presented in Table 2), while treating a car wash wastewater [34]. No significant difference was observed in the results by the increase in the inter-electrode distance from 1 to 3 cm, which led the authors to conclude that, despite this being an effective parameter in the electrocoagulation process, it is insignificant in the combined metal-activated persulfate with simultaneous electrocoagulation. 

The most common persulfate source in the studies presented in Table 2 is PDS, although some studies utilized PMS, and in some others both oxidants were compared. Varank et al. [30] treated a nanofiltration concentrate from SLL utilizing PMS and PDS and observed that, although both PS sources were effective in the treatment of the leachate concentrate, the best results were attained with PMS. Conversely, in a similar study, Guvenc and co-workers [11] found slightly higher TOC removals when PDS was employed, although the use of PMS led to a significant decrease in the energy consumption (PDS—5.81 kWh m^−3^ and PMS—1.87 kWh m^−3^). According to the authors, this lower energy consumption is related to a lower reaction time, which makes the PMS process more advantageous. It is noteworthy that PMS is a more expensive reagent that PDS, since it is used as a triple salt to be stable.

Persulfate dosage is a key parameter in PS electro-activation through sacrificial anodes and, in the studies developed, different PS/COD mass ratios have been applied, in the range of 0.2 to 6.5. In a study performed by Bashir et al. [33], with a palm oil mill effluent, it was found that, when PDS concentration increased from 1 to 2.76 g L^−1^, COD removal increased, but, when PDS concentration increased from 2.76 to 8 g L^−1^, a decrease in COD removal was observed. According to the authors, a higher PDS amount leads to higher H_2_O_2_ formation, which consumes dichromate ion during COD determination (Equations (31) and (32)), increasing the final COD value. A similar result was obtained when treating a pulp and paper wastewater by electro-PMS process with iron electrodes, with COD removals of 18, 41, and 40% for PMS concentrations of 226, 678, and 904 mg L^−1^, respectively [12]. According to these authors, the PMS excess can act as a sulfate radical scavenger (Equation (33)). This observation about the negative effect of a PMS excess was also observed during single electrolytic activation of persulfate [27].

S_2_O_8_^2^^−^ + 2H_2_O → H_2_O_2_ + 2HSO_4_^−^, (31)

Cr_2_O_7_^2^^−^ + 3H_2_O_2_ + 8H^+^ → 2Cr^3+^ + 3O_2_ + 7H_2_O, (32)

HSO_5_^−^ + SO_4_^•−^ → SO_4_^2^^−^ + SO_5_^•−^ + H^+^. (33)

Varank and collaborators [29,30] performed the electro-persulfate assisted degradation of SLL concentrates, with different initial CODs, utilizing different PMS/COD and PDS/COD ratios. For an initial COD of 6.2 g L^−1^, the most favorable PMS/COD and PDS/COD ratios for COD removal were 2.5 and 1.9, respectively [30]. When initial COD was about 5.1–5.4 g L^−1^, the most advantageous PDS/COD ratios for COD and color removal were 1.72 and 3.5, respectively [29]. In a similar study, Guvenc et al. [11] utilized PMS and PDS with oxidant/COD ratio between 0.5 and 2.5, and obtained the best TOC removal for oxidant/COD ratio of 2. Onn et al. [28] have treated a landfill leachate using PDS/COD values between 1 and 6 and, applying ANOVA methodology, color and COD removals were maximized for an oxidant/COD ratio of 4. The treatment of an effluent from paper industry was performed by Varank et al. [15]. PDS/COD ratios from 0.5 to 6.5 were assayed, using iron or aluminum consumable electrodes. Under the optimum conditions, the COD removal efficiency, predicted by surface response model, was 63.5 and 72.8% for iron electrodes (PDS/COD = 1.25, I = 4.14 A, pH = 6 and reaction time 5 min) and for aluminum electrodes (PDS/COD = 0.5, I = 4.25 A, pH = 7.25 and time reaction 25 min), respectively. For most of the referred studies, high PDS/COD may show a negative impact in COD removal and, according to the authors, this must be due to sulfate radical recombination to restore peroxydisulfate (Equation (27)). 

In a study with a biodiesel wastewater, the influence of PDS/COD ratio between 1 and 5 was assessed, aiming to maximize the removal of COD, oil-grease, and volatile fatty acids, and to minimize the costs [31]. For the optimum experimental conditions, an oil grease removal of 97.2% was obtained, using a PDS/COD ratio of 4.4. For the same effluent, total suspended solids (TSS) removal was evaluated [32] and 86.6% TSS removal was obtained for the optimum conditions, with a PDS/COD ratio of 1. Once again, it was observed that the increase in the oxidant concentration is beneficial until an optimum value, which depends on the effluent type and the parameter to be optimized.

Durna and Genç [34] have treated a car wash wastewater by PDS electro-activation through aluminum anodes, using microwaves (MW) to pre-activate PDS, and adding O_3_ during the experiments. PDS/COD ratios between 1.5 and 3 were assayed at very low applied current densities (0.0159 or 0.0319 mA cm^−2^), when compared to others studies. The COD removal for MW + the electro-persulfate process was 80% at the optimized experimental conditions (pre-treatment with MW, with power at 567 W during 30 min, as well as a PS dosage of 2.5 g L^−1^, current density of 0.0159 mA cm^−2^, 20 min, 1-cm inter electrode distance and pH 9). For the electro-persulfate+O_3_ process, the COD removal was 56% at the optimized experimental conditions (PS dosage 1.25 g L^−1^, current density 0.0319 mA cm^−2^, 50 min, 3-cm inter electrode distance, pH 9, ozone dose 0.6 g h^−1^, and ozone flow 2 L min^−1^). The best result for the MW + electro-persulfate process was obtained at the highest PDS dosage tested and, for the electro-persulfate + O_3_ process, the optimum PDS dosage was the lowest one.

Current density is another important variable that influences pollutants degradation rate, since the increase in current promotes the increased Fe^2+^ production and persulfate activation, and more radicals are available to attack organic pollutants. Although the different works performed, with the objective of studying the influence of current density on the COD removal rate, very different current densities were assayed, most of them have found an optimum current value that, in general, was not the highest assayed [11,12,13,28,29,30,31,32,33,34]. In fact, higher currents may slow down the organics degradation rate, since an increase in current leads to an increase in ferrous ion in solution, which can act as a sulfate radical scavenger (Equation (34)).

Fe^2+^ + SO_4_^•−^ → Fe^3+^ + SO_4_^2^^−^. (34)

As can be seen in Table 2, the natural pH of the studied effluents varies between 5 and 8.5. In some studies, the influence of this parameter in the efficiency of the treatment was evaluated and the experiments were performed at different adjusted pH values. Ahmadi and Ghanbari [13] have performed experiments at pH of 5, 7, and 9, and concluded that the pH effect was negligible in the range from 5 to 9, meaning that the COD removal did not depend on pH. According to the authors, a possible explanation for this is: (i) coagulation with iron is effective for the pH range of 4 to 9; and (ii) sulfate radical can degrade the organic matter in acidic condition, whereas in the presence of hydroxyl ion (pH > 7), HO^•^ is produced (Equation (35)), assuring that oxidants are always present, no matter solution pH.

SO_4_^•−^ + HO^−^ → SO_4_^2^^−^ + HO^•^. (35)

In the work developed by Bashir and co-workers [33], when treating a palm oil mill wastewater, COD removal increased when pH decreased from 5 to 3. According to the authors, at lower pH, the oxygen evolution overpotential increases, decreasing the competition between the organic matter oxidation and oxygen evolution reaction.

During the treatment of a leachate nanofiltration concentrate, Varank et al. [29] studied the influence of pH (from 3 to 9) on COD and color removals and, similarly to Ahmadi and Ghanbari [13], observed that the influence of pH was not significant. Nevertheless, the best results for COD and color removals were obtained at a pH of 5. In a similar study, Varank et al. [30] concluded that the optimum pH with PMS was 6.4 and with PDS was 5.1. Guvenk et al. [11] also varied the initial pH (from 3 to 7) of a leachate nanofiltration concentrate and obtained a maximum TOC removal and minimum energy consumption at a pH of 5.64 for PMS and a pH of 4.55 when PDS was used as persulfate source. Varank et al. [15] changed pH between 6 and 12, while studying the treatment of a paper industry wastewater, and concluded that the optimum pH with Al electrodes was 7.25 and, with Fe electrodes, was 6. Thus, in conclusion, it may be pointed out that, although the optimum pH depends on the organic load and type of effluent, the ideal pH is acidic and presents values between 5 and 6.

#### 3.2.2. Persulfate Electro-Activation with Metal Ions Addition

Persulfate electro-activation with metal ions addition was applied in the treatment of SLL [35], SLL nanofiltration concentrate [36], mixed industrial wastewater [37], olive mill wastewater [38], and dyeing wastewater [24]. Table 3 summarizes the conditions used in these studies, as well as the main results obtained. In all the studies presented, the persulfate source utilized was PDS and the main variables evaluated were PDS concentration, added iron concentration, applied current, and initial pH.

As described in the previous section, pH plays an important role in the combined electrolytic and metal activation of persulfate. In the study performed by Zhang et al. [35], while treating a sanitary landfill leachate, it was observed that initial pH adjustment, from alkaline to acid, resulted in a marked increase in the COD removal during the first hour of assay. According to the authors, at alkaline initial pH, the buffer capacity of the SLL prevented the pH from falling lower than 7, causing a deactivation of the ferrous ion into ferric hydroxo complexes. Despite this, the authors noticed that the formation of ferric-oxyhydroxides promoted the removal of the organics from the SLL by coagulation and, consequently, the overall COD removal was considerably improved. According to Chanikya et al. [24], which studied the effect of the initial pH on the electro-persulfate treatment efficiency of a dyeing wastewater, at acidic pH values, ferrous ion is the main activator for PS ions. However, at neutral and alkaline conditions, since ferrous ions exist in its insoluble hydroxide forms, it is inefficient for PS activation. Thus, the PS activation, at higher pH conditions, mainly occurs by cathodic reduction. Nonetheless, the authors observed that the COD removal, after 60 min of electrolysis, remained almost the same in all tested pH conditions and attributed that to the hydroxyl radicals generated by the reaction between the sulfate radical and the hydroxyl ion, at alkaline conditions (Equation (35)).

Iron concentration is another key factor in the combined electrolytic and metal activation of persulfate. According to Zhang et al. [35], the increase in iron ion dosage enhances the available Fe^2+^ concentration, resulting in an increased PDS activation. In the study performed by these authors, during the treatment of a SLL, COD removal by oxidation increased from 33.5% to 40.1%, when Fe^2+^ concentration rose from 7.81 to 15.6 mM. However, when Fe^2+^ concentration was further increased to 31.2 mM, the COD removal by oxidation decreased to 23.1%, which was explained by the scavenging reaction between Fe^2+^ and SO_4_^•−^, promoted by the excess amount of Fe^2+^ in solution (Equation (34)). Despite this, the authors observed that the increase in iron dosage increases the production of ferric-oxyhydroxides during the neutralization stage, which favored the COD removal by coagulation. Similar results and conclusions were attained in the study performed by Cui et al. [36], where an increase in Fe^3+^ dosage, from 3.75 to 15 mM, resulted in an increase in COD removal from 21% to 55%, with a variation in COD removal by oxidation from 18% to 28% and a variation in COD removal by coagulation from 3% to 27%.

Among the several operational parameters that affect the effectiveness of the electro-persulfate treatment, PS concentration is one of the major factors that limit the performance of the process [38]. To evaluate the influence of PDS concentration on the COD removal from a SLL, Zhang et al. [35] performed experiments where the added PDS concentration was varied from 15.6 to 62.5 mM, and observed an increase in COD removal with PDS concentration. However, this increase in COD removal was not proportional to the increase in PDS concentration, since, according to the authors, the increasing PDS concentration results in the enhancement of the side reaction between S_2_O_8_^2^^−^ and SO_4_^•−^ (Equation (26)). In a similar study performed by Cui et al. [36], it was observed that the COD removal increased from 41% to 55% with the increase in PDS concentration from 18.75 to 37.5 mM. However, a further increase in PDS concentration resulted in a decrease in COD removal, which can be explained, according to the authors, by the enhanced PDS reduction at the cathode, which inhibited the Fe^3+^ reduction, required for the effective PDS activation. Identical results, but different explanation, were described by Popat et al. [37]. While treating a mixed industrial wastewater, the authors observed that the COD removal increased linearly with PDS dosage until an optimum value, from which an opposite trend was observed. According to the authors, an excessive increase in oxidizing agent concentration, above an optimum level, results in less availability of sulfate radicals with time, due to the enhancement of the reactions described by the Equations (34) and (36).

SO_4_^•−^ + SO_4_^•−^ → 2SO_4_^2^^−^. (36)

From the results presented, it can be concluded that there is an optimal PS concentration for the efficient electro-persulfate process through the addition of metal ions, above which sulfate radicals became less available for the pollutants oxidation. Furthermore, a study performed by Görmez et al. [38] discloses a synergistic effect of PS and iron concentrations on COD removal.

Since applied current is a key operational parameter in electrochemical processes, different studies have assessed its influence on the performance of the persulfate electro-activation with the addition of metal ions. It is expected that an increase in applied current leads to a more rapid regeneration of ferrous ion via cathodic reduction reaction, enhancing the PS activation. However, the results obtained in different studies show that the increase in PS activation with applied current is not linear. In the treatment of SLL through an electro/Fe^2+^/PDS process, Zhang et al. [35] observed that an increase in current density, from 6.94 to 13.89 mA cm^−2^, resulted in increased PDS decomposition percentages, from 66.2% to 81.6%, and increased COD removals by oxidation from 22.6% to 40.1%. However, when the current density was increased from 13.89 to 27.78 mA cm^−2^, the remained percentage of PDS was almost the same as that at 13.89 mA cm^−2^, and COD removal by oxidation only increased from 40.1% to 42.2%. According to the authors, this is due to the enhancement of side reactions, such as oxygen and hydrogen evolution, that inhibit the main reactions, such as the electro-regeneration of ferrous ion from ferric ion, and, consequently, the activation of PDS. The authors also noticed that the COD removal by coagulation decreased with current density, probably due to the decomposition of the Fe(OH)_n_ flock structures, caused by the production of oxygen and hydrogen. Similar results and conclusions were obtained from the study performed by Cui et al. [36] on the treatment of a SLL nanofiltration concentrate. In a different study, while treating a dyeing wastewater, Chanikya et al. [24] evaluated the effect of applied voltage (3, 5, and 9 V) and observed that, although insignificant changes in COD removal were observed at 5 V, compared to 3 V, a significant increase in COD removal occurred at 9 V, reaching 89.4% COD removal. On the other hand, specific energy consumptions increased with the applied voltage, which was ascribed to the reduction in ICE values with increasing voltage, due to hydrogen evolution reactions and other parasitic reactions. The authors have concluded that, although higher applied voltage attained higher COD removal, it is not the best choice from the economic point of view. The same conclusion was attained by Görmez et al. [38], while treating an olive mill wastewater by electro/Fe^2+^/PDS process.

#### 3.2.3. Persulfate Electro-Activation Using Metal-Based Catalysts

The use of metal-based catalysts in the electro-persulfate treatment of SLL [39,40], SLL nanofiltration concentrate [16], and washing machine effluent [26] has been reported, and a brief summary of these studies is presented in Table 4. 

Aiming to treat a SLL nanofiltration concentrate, Wang et al. [16] studied the performance of an electro-PMS process in the absence and presence of manganese ferrite nanoparticles, MnFe_2_O_4_, or embed MnFe_2_O_4_ nanoparticles on a nitrogen-doped reduced graphene oxide matrix (NrGO), NrGO-MnFe_2_O_4_. Additionally, the authors compared the performance of these electro-PMS processes with single electrolysis (without PMS), single PMS, PMS/NrGO-MnFe_2_O_4_, and single NrGO-MnFe_2_O_4_. For the same experimental conditions, after a 2-h reaction, the COD removal achieved in the different treatment systems followed the order: single NrGO-MnFe_2_O_4_ (9.21%) < single PMS (12.17%) < single electrolysis (30.12%) < PMS/NrGO-MnFe_2_O_4_ (~32%) < electro/PMS (42.33%) < electro/PMS/MnFe_2_O_4_ (~55%) < electro/PMS/NrGO-MnFe_2_O_4_ (~72%). The better performance of the electro/PMS/NrGO-MnFe_2_O_4_ system was attributed to: (a) the doping of nitrogen atoms forms more active centers, which can activate PMS; (b) NrGO scaffolds significantly promote the accessibility and adsorption of substrates to active centers; (c) MnFe_2_O_4_ particles are more evenly distributed on the NrGO surface, accelerating its activation on PMS; and (d) strong electrical conductivity accelerates electron transfer between MnFe_2_O_4_ and the electrode. The influence of pH, catalyst dosage, PMS dosage, current density, and inter-electrode gap, on the SLL concentrate treatment by electro/PMS/NrGO-MnFe_2_O_4_ process was also assessed (Table 4).

The application of a three-dimensional electrode, created by using Fe/C granules, which were suspended between the cathode and the anode, to treat a SLL by electro-PDS process, was studied by Yu et al. [39], and the results were compared with different treatment systems: single electrolysis (without PDS or Fe/C granules), electrolysis with PDS, electrolysis with Fe/C granules, and PDS with Fe/C granules (without electrolysis). The lowest COD removal (4%), after a 2-h assay, was attained by the PDS with Fe/C granules system, without electrolysis, since, according to the authors, the iron-carbon-activated persulfate is unable to produce a large number of free radicals to remove the organic pollutants from the SLL. For the electrochemical treatment systems, the COD removal followed the order: single electrolysis (46.9%) < electrolysis with Fe/C granules (50.4%) < electrolysis with PDS (53.7%) < electrolysis with PDS and Fe/C granules (72.91%).

These results were further compared with a homogenous Fe^2+^ system, by replacing the Fe/C granules by an iron salt, and it was found that, although the homogenous Fe^2+^ system presented better performance than the electrolysis with PDS, the best results were attained by the electrolysis with PDS and Fe/C granules. The reusability and stability of the Fe/C granules were assessed. Six sequential experiments were performed and all resulted in COD removals higher than 65%, with 10% variation between experiments. Although some Fe^0^ was lost during the assays, ranging from 6.21 to 5.60 mg L^−1^, the element composition of both fresh and used materials showed that the main elements contained in the fresh material were similar to those in the used material.

Zhang et al. [40] developed a combined PDS/iron-carbon microelectrolysis system (PDS-ICME) to treat a SLL. The optimal parameters, including Na_2_S_2_O_8_ dosage (85 mM), Fe-C ratio (3), and initial pH (7), were determined by response surface methodology, in terms of the highest COD removal (62.91% after a 1-h assay). According to the authors, at neutral pH, Fe^2+^ is easily converted into Fe^3+^, which combines with hydroxide to form Fe(OH)^2+^, Fe(OH)_2_^+^, and Fe(OH)_3_, being the SLL pollutants removed by flocculation and precipitation. Moreover, electron spin resonance spectrum investigation demonstrated that the signal intensity of free radicals (SO_4_^•−^ and HO^•^) was highest under neutral initial pH conditions, indicating that the organic matter in SLL was degraded through the oxidation by SO_4_^•−^ and HO^•^. In fact, three-dimensional fluorescence characterization results showed that the PDS-ICME process can effectively degrade the high molecular weight SLL components, such as humic acid substances. 

Ghanbari and Martínez-Huitle [26] treated a washing machine effluent by electro-PMS coupled with magnetite (Fe_3_O_4_) nanoparticles, and compared the obtained results with different systems, operated under the same conditions. While single electro-PMS (without Fe_3_O_4_) led to 32.7% COD removal, after a 180-min reaction, electro-PMS with magnetite nanoparticles led to 74.4% COD removal. According to the authors, in this latter process, PMS is additionally activated by the magnetite nanoparticles, according to Equations (17) and (19). The influence of solution pH, current density, magnetite nanoparticles dosage, and PMS concentration, on the performance of the electro-PMS process coupled with magnetite nanoparticles, was evaluated. The optimal pH was found to be 5. Regarding the applied current density, an increase in COD removal with current density, from 10 to 30 mA cm^−2^, was observed, but, for higher applied currents, there was no significant augment in COD removal. Magnetite nanoparticles dosage and PMS concentration also played an important role on COD removal. A considerable increase in COD removal was observed when Fe_3_O_4_ dosage was increased from 25 to 100 mg L^−1^, which, according to the authors, was due to more available Fe_3_O_4_ sites for the reaction with PMS, to generate free radicals. However, above 100 mg L^−1^, the trend of COD removal was steady state, which was ascribed to the nanoparticles agglomeration and consequent reduced reactional surface. Regarding PMS concentration, a similar behavior was observed: COD removal increased with PMS concentration, from 0.5 mM to 3 mM, but decreased at the PMS concentration of 4 mM. The authors ascribed this decrease in COD removal at higher PMS concentration to the reactions between PMS and sulfate radical and hydroxyl radical (Equations (33) and (37) to (39)), which remove free radicals from the chain reactions of organic pollutants degradation and produce weaker oxidants.

HSO_5_^−^ + HO^•^ → SO_5_^•−^ + H_2_O, (37)

HSO_5_^−^ + HO^•^ → HO_2_^•^ + SO_4_^2^^−^ + H^+^, (38)

HSO_5_^−^ + SO_4_^•−^ + H_2_O → HO_2_^•^ + 2SO_4_^2^^−^ + 2H^+^. (39)

Magnetite nanoparticles reusability and stability were also evaluated. After each experiment, the catalyst was, according to the authors, effortlessly and quickly recovered by an external magnet. A decrease in the magnetite nanoparticles catalytic activity was observed after four cycles of its usage without any regeneration method. COD removal was reduced from 74.4% to 66%. Authors ascribed this decrease in catalytic activity to the: (i) corrosion of the magnetite nanoparticles surface by PMS, a strong oxidant, which resulted in the reduction in the magnetite active sites; (ii) existence in the effluent of many organic compounds, oxidized forms, and ions that induced the magnetite deactivation; and (iii) aggregation of these substances on the magnetite surface. The stability of magnetite nanoparticles was evaluated by iron measurements in the effluent, after each reusability experiment. Iron concentration in the effluent increased from 0.11 mg L^−1^ (before treatment) to 0.48 mg L^−1^ after four usages of magnetite nanoparticles.

Although the reusability and stability of the catalysts were only evaluated in some studies, it is one of the most important criteria for the practical application of persulfate electro-activation processes using metal-based catalysts. A good catalyst should be resistant to corrosion and oxidation and should not present reduced catalytic activity when reused [26].

### 3.3. Electro-Persulfate Processes Involving Co-Activation by Irradiation or Heat

Among the different studies involving the application of electro-persulfate processes in the treatment of complex wastewater matrices, there are a few that combined the electrochemical PS activation with irradiation or heat as co-activators [13,20,22,23,26,41,42,43]. Table 5 summarizes the conditions used in the electro-persulfate studies involving co-activation by irradiation or heat, as well as the main results obtained.

Electro-persulfate processes involving co-activation by ultrasound radiation were applied in the treatment of petrochemical [22,41] and textile [42,43] wastewaters. Ahmadi et al. [41] assessed the influence of different variables, such as pH, applied voltage, and US power, in a US-assisted electro-Fenton process for the treatment of saline petrochemical wastewater. At the optimized operational conditions, the effect of PDS concentration on the COD removal was studied. The authors have reported that an increase in PDS concentration, from 0 to 0.75 mM, resulted in an increase in COD removal from 80.2% to 91.7%. However, a further increase in PDS concentration to 1 mM resulted in a slight decrease in COD removal. In this combined process, both the continuous production of iron ions in the anode, and the application of US radiation, are responsible for the PDS activation and sulfate radical formation (Equations (14) and (22), respectively). Therefore, the initial increase in PDS concentration leads to the formation of more sulfate radicals to partake in wastewater treatment and COD removal. However, with excess PDS ions, an adverse effect starts to occur, as they act as sulfate radical scavengers, hindering the process and decreasing its efficiency (Equation (26)).

Yousefi et al. [22] also studied the influence of different experimental variables on the sonoelectro-activated persulfate oxidation of a saline petrochemical wastewater. A similar behavior was reported for the increase in the PDS concentration, with the identification of an optimal concentration (20 mM), followed by a loss in efficiency for higher PDS concentrations. The influence of the US power in the treatment performance was also assessed. The increase in this parameter, from 100 to 300 W, resulted in an increased COD removal, from 64.8% to 73.9%. According to the authors, the increase in US intensity: (i) generates microstreaming and cavitation bubble, helping the solution mixing, by eliminating mass transfer resistance, and promoting the cleaning of the catalyst’s surface, for further reactions to occur; and (ii) boosts the decomposition of the PDS ions and the production of hydroxyl and sulfate radicals. In fact, the addition of US to the electro-activated persulfate process resulted in an increase in COD removal from 68.97% to 82.31%, showing the synergistic effect of the combined process. Additionally, the authors have reported that an increase in temperature, from 20 °C to 60 °C, resulted in an increased COD removal, from 82.3% to 91.2%.

In the treatment of a real textile wastewater by a sonoelectrochemical process, Johin et al. [42] studied the influence of different operational parameters, such as initial pH, US power, and voltage, in the degradation of aqueous solutions of Reactive Black 5 dye.

Two electrolytes, sodium peroxydisulfate and manganese sulfate, were also applied, and different concentrations were tested. During the sonoelectrochemical process, PDS can dissociate into two sulfate radical anions and further produce hydroxyl radicals, with both species being strong oxidizing agents (Equations (22) and (8), respectively). For manganese sulfate, the application of US promotes the split of manganese cation and sulfate radical anion (Equation (40)), with the divalent manganese ions playing an important role in the formation of sulfate radicals, by reacting with the PDS (Equation (41)).

MnSO_4_ + US → Mn^2+^ + SO_4_^•−^, (40)

S_2_O_8_^2^^−^ + Mn^2+^ → Mn^3+^ + SO_4_^2^^−^ + SO_4_^•−^. (41)

The optimized operational conditions (100 mg L^−1^ of sodium peroxydisulfate, 75 mg L^−1^ of manganese sulfate, pH of 8.05, US power of 44 W, and applied potential of 20 V) were applied to the real wastewater and a TOC removal of 90 % was obtained after 60-min treatment. Utilizing the real effluent, the effect of the US radiation was evaluated at an applied potential of 8 V. When compared to the experiments without US radiation, a slight increase in TOC removal was observed for the combined process, showing the effect of the sonoelectrochemical treatment using MnSO_4_/Na_2_S_2_O_8_ electrolytes.

Jorfi and Ghaedrahmat [43] also evaluated the electro/US/PDS process efficiency in the treatment of a textile wastewater. Parameters such as pH, electrolyte (Na_2_SO_4_) concentration, applied voltage, PDS concentration, and US power were studied, and the optimal conditions determined. Under those optimal conditions (pH = 5, [PDS] = 0.5 mM, [Na_2_SO_4_] = 6 g L^−1^, US power = 300 W) a COD removal of 96% was achieved after a 90-min assay. Given that a consumable iron anode was employed, it should be noticed that Fe^2+^ was present in the solution, contributing to the PDS activation and sulfate radical formation. 

The electro-persulfate process involving co-activation by UV irradiation was applied in the treatment of a washing machine effluent [26], a landfill leachate [20] and a greywater [13]. Ghanbari and Martínez-Huitle [26] applied a photoelectro-Fenton (PEF) process combined with PMS for the treatment of washing machine effluent. A heterogeneous iron-based catalyst (Fe_3_O_4_) was used, under a UVC lamp (4W, 254 nm) with UV irradiance of 1.02 mW cm^−2^. Under the optimal conditions, a COD removal of 99.5% was obtained. In the absence of UV irradiation, the COD removal was only 74.4%. According to the authors, UV irradiation decomposed the electrogenerated hydrogen peroxide, generating hydroxyl radicals that additionally oxidized the organic compounds. Furthermore, UV irradiation played as catalyst regenerator, in the regeneration of Fe(III) to Fe(II) on the catalyst surface, and as PMS activator through Equation (21). In this way, PMS was activated through three methods, i.e., electrolysis, transition metal, and photolysis.

For the treatment of a landfill leachate, Silveira et al. [20] applied an electro-persulfate process combined with 30 W UV-LED radiation, and added ilmenite (FeTiO_3_) as a Fe(II) source. A continuous batch-recirculated system was employed, and different experimental conditions, such as current density (50–200 mA cm^−2^), PDS concentration (46.8–234 mM) and ilmenite concentration (0.5–1.5 g L^−1^), were assayed. Regarding the influence of the ilmenite concentration in the treatment performance, it was reported that an increase from 0.5 to 1 g L^−1^ resulted in an enhanced TOC removal. However, a further increase to 1.5 g L^−1^ showed to be detrimental for the process efficiency, due to the increase in turbidity that hindered the UV radiation penetration through the solution. Since, during this treatment process, the pH of the leachate decreased from 8.5 to below 3 (after a 300-min assay), the possibility to apply a consecutive Fenton-like oxidation, by the addition of H_2_O_2_, was evaluated. Stoichiometric quantities of H_2_O_2_ were added to the leachate (2.12 g/g COD) in three steps (at 300, 360, and 420 min), to reduce the scavenging reactions with Fe^2+^, H_2_O_2_, and HO_x_^•^. The efficiency of this combined treatment was above 90%, in terms of TOC removal, after 480 min.

Ahmadi et al. [13] studied the application of an electro-persulfate process combined with two 6-W UVC lamps for the treatment of greywater. The COD removal optimization was performed using the Box–Behnken design as a response surface methodology, with four independent variables: electrolysis time, current density, PDS dosage, and pH. An improvement in the electro-persulfate process performance by the addition of the UV irradiation was observed, with an increase in COD removal from 68 to 77 %. This improvement was ascribed to the direct activation of PDS by UV (Equation (22)) and to the regeneration of the ferrous ion, obtained from the anodic dissolution of the consumable iron anode, by UV, with consequent hydroxyl radical production (Equation (42)).

Fe(OH)^2+^ + UV → Fe^2+^ + OH^•^. (42)

Xue and collaborators [23] developed a heat-assisted electro/PDS/Fe^2+^ process for the treatment of a landfill leachate nanofiltration concentrate. The process was carried in a H-type reactor, divided into two chambers by a proton exchange membrane. A two-stage procedure was employed, consisting in 15 min of anodic reaction, followed by 105 min of cathodic reaction. At the optimized experimental conditions, the combined heat/electro/PDS/Fe^2+^ process resulted in a COD removal of 87%, which was considerably higher than the COD removal attained by the heat/PDS process (43%). The influence of PDS concentration (37.5–150 mM), applied current (40–160 mA), and Fe^3+^ dosage (3.75–15 mM) were assessed. For PDS and Fe^3+^ concentrations of 75 and 15 mM, respectively, at an applied current of 80 mA, the raise of the temperature from 60 °C to 80 °C resulted in an increased COD removal from ~50% to 87%. However, no significant increase in COD removal was observed when the temperature was further raised to 90 °C.

## 4. Major Challenges and Future Prospects

The literature review on the application of electro-activated persulfate processes in the treatment of complex matrix effluents validates this technology as an efficient way of reducing the pollutant load and recalcitrant constituents in this kind of effluents. Nevertheless, some challenges remain, namely those concerning efficiency and cost-effectiveness.

Sulfate radical-based electrochemical processes can be accomplished either by adding persulfate to the effluent to be treated, or by producing it in situ, in effluents containing sulfate, through sulfate direct oxidation or indirect oxidation with hydroxyl radicals. When externally added, the amount of persulfate is one of the most relevant variables to be optimized. Several authors have evaluated this parameter, and, in general, have concluded that there is an optimum PS/COD ratio regarding the organic load removal, which may not coincide with the PS/COD ratio for the color removal, for instance. This ratio may be presented in different ways (mass or molar ratio or mass percentage), and this lack of uniformization makes it difficult to compare results. This type of information would be very useful to systematize results and make them more appropriate for future applications at an industrial level.

Current density/potential is another parameter of key importance in the electrolytic activation of persulfate, since it directly affects persulfate activation and regeneration, sulfate and hydroxyl radicals formation and current efficiency, thus being highly reflected in the treatment cost. So, a proper steadiness must be attained prior to the process scale-up.

The anode material plays an important role too, namely in persulfate and sulfate radical production/regeneration, by sulfate anodic oxidation, and in hydroxyl radical formation and oxygen evolution. BDD has been described in the literature as one of the most efficient anode materials for electro-persulfate activation, due to its unique physical-chemical properties, besides the large potential window, that makes it an appropriate material to regenerate PS from sulfate, form hydroxyl and sulfate radicals, and delay oxygen evolution. 

Still, mass transfer limitations have been reported in processes with single electrolytic persulfate activation, since the reactions mainly occur at the electrodes surface, increasing treatment time and energy consumption. To overcome this drawback, PS electrochemical activation has been combined with in bulk PS activation methods, namely with those presented in Table 6. Although the PS activation efficiency is usually enhanced by the combination of electrochemical activation with other PS activation methods, there are some aspects that need to be taking into account when considering a hybrid PS electro-activation process (Table 6). Another possibility to solve the problem raised by the limitation in mass transfer is to improve reactor configuration and, recently, reactive electrochemical membranes are being utilized as a flow-through electrode. These electrochemical membranes significantly increase the active surface area, while enhancing pollutants mass transport by convection [44,45,46].

Although the works described in the literature have studied the influence of many different experimental aspects of the electro-activated persulfate processes, toxicity assessment of the treated effluents has been disregarded and insufficiently addressed in the application of electro-persulfate processes. This is an important field to explore since by-products toxicity may cause serious environmental concerns. A recent review focus on the different parameters influencing by-products toxicity, and a comparison of the effect of the experimental conditions and the type of radicals involved in the AOPs, including hydroxyl and sulfate radicals, is made [47]. However, as it became clear during the present review, several radical species, as well as other oxidants (active chlorine species, hydrogen peroxide, etc.), may be involved in the electro-persulfate processes. Thus, when considering complex wastewater matrices with diversified composition, a toxicity evaluation is mandatory if the process is intended to be applied at an industrial scale. In comparison with other AOPs, an analysis on the toxicological effects would be desirable, but is currently not possible due to the lack of literature. 

The addition of persulfate leads to a significant sulfate concentration, which can be a problem in the treated effluent. Although electro-persulfate processes may enable persulfate regeneration, thus reducing the amount required for the effective treatment of the effluents, final sulfate content in the treated effluents must be properly addressed prior to make it a proper industrial offer. 

Finally, the problem associated with all electrochemical processes is the energy consumption. Besides the integration of electrochemical persulfate activation with other activation methods, which decreases the treatment costs, the integration with different treatment technologies and the use of renewable energy sources, to power the electrochemical system, are possible solutions to overcome this drawback. In fact, with the many green energy options available nowadays, energy consumption must become a minor drawback in the near future.

Despite the challenges that remain in optimizing the electro-persulfate processes for more effective and cost-efficient treatment in practical systems, the application of these processes as end-of-line technology is feasible, in centralized or decentralized wastewater treatment systems. As in the generality of the electrochemical processes, electro-persulfate processes efficiency increases with the wastewater organic load, meaning that these processes are preferred for decentralized industrial wastewater treatment systems, with high organic load and low effluent volume. Nonetheless, electro-persulfate processes were applied to effluents with low organic load with success [8,24,25,26,33].

## Figures and Tables

**Table 1:** Summary of research results previously reported for single electrolytic activation of persulfate in the treatment of complex wastewaters.

Type of Effluent	Anode/ Cathode	PS Source	[PS]_added_	Applied Current	T/°C	Time/h	Treated Volume/L	pH_0_	[Organic Load]_0_/mg L^−1^		Organic Load Removal/%		Energy Consumption/kWh m^−3^ Order^−1^	Reference
									COD	TOC	COD	TOC		
Dyeing wastewater	(Pt-Ti)/ Fe	PDS	0 g L^−1^	3 V	NS	1	1	6	237	NS	56	NS	NS	[24]
			0.5 g L^−1^								76			
			1 g L^−1^								72			
Industrial effluent containing DNTs	Pt/ Pt	PDS	1 wt%	3 V	30	8	0.45	0.5	NS	300	NS	20	NS	[25]
				4 V								35		
				5 V								48		
				6 V								70		
					35							73		
					40							75		
					45							79		
			0.7 wt%									70		
			1.3 wt%									84		
			1.7 wt%									95		
			1 wt%					1				74		
			1 wt%					2				70		
			1 wt%					3				66		
Washing machine effluent	Pt/ Graphite felt	PMS	2 mM	30 mA cm^−2^	NS	3	0.2	6.7	NS	202	NS	32.7	NS	[26]
Oil sands process water	BDD/Stainless steel	NA	NA	5 mA cm^−2^	23	6	0.45	8.6	NS	73.8	NS	95 ^1^	NS	[8]
				10 mA cm^−2^								100 ^1^		
				20 mA cm^−2^								100 ^1^		
				30 mA cm^−2^								100 ^1^		
Cyanide-containing wastewater	BDD/ Stainless steel	PDS	0.1 M	10 mA cm^−2^	20	24	0.5	5.6	11290	4456	88.8	82.8	60.6	[27]
				20 mA cm^−2^							99.5	87 (16 h)	62.5	
				30 mA cm^−2^							99.8	98.2 (16 h)	84.8	
				10 mA cm^−2^				2.1			90.1	86.3	56.7	
								11.8			73	61	122.5	
					40			5.6			95.8	87.8	41.6	
					50						97	90	30.1	

NA-Not applied; NS-Not specified. ^1^ Value obtained indirectly from data presented in the paper or from a figure.

**Table 2:** Summary of research results previously reported for persulfate electro-activation through sacrificial anodes in the treatment of complex wastewaters.

Type of Effluent	Anode/Cathode	PS Source	[PS]_added_	Applied Current	Electrolysis Time/Min	Treated Volume/L	pH_0_	[Organic Load]_0_/mg L^−1^		Organic Load Removal/%		Energy Consumption	Reference
								COD	TOC	COD	TOC		
Landfill leachate	Al/Al	PDS	0.5 g L^−1^	10 mA cm^−2^	80	NS	4	492	NS	31.52	NS	NS	[28]
				60 mA cm^−2^						36.39	NS	NS	
			3 g L^−1^	10 mA cm^−2^						17.27	NS	NS	
				60 mA cm^−2^						28.01	NS	NS	
			0.88 g L^−1^	44.66 mA cm^−2^	68.3					45.70	NS	NS	
Landfill leachate concentrate	Fe/Fe	PDS	PDS/COD ratio 1.72	1.26 A	34.8	0.5	5	5250	NS	72.6	NS	NS	[29]
Landfill leachate concentrate	Fe/Fe	PMS	PS/COD ratio 2.5	1.8 A	35.9	1	6.4	6200	NS	84.2	NS	NS	[30]
		PDS	PS/COD ratio 1.9	2.1 A	32.3		5.1			79.6	NS	NS	
Landfill leachate concentrate	Fe/Fe	PMS	PS/COD ratio 2	1 A	15	NS	5.64	5250	NS	NS	56.91	1.87 kWh m^−3^	[11]
		PDS			33.8		4.55			NS	58.43	5.81 kWh m^−3^	
Biodiesel wastewater	Fe/Fe	PDS	PDS/COD ratio 1	1 A	15.6	0.5	2	95000	1300 (TSS)	NS	90.6 (TSS)	NS	[31]
Biodiesel wastewater	Fe/Fe	PDS	PDS/COD ratio 4.4	4 A	15	0.5	2	95488	NS	49.0	NS	NS	[32]
Pulp and paper wastewater	Fe/Fe	PMS	0 mM	0.5 mA cm^−2^	60	0.5	4.9	585 ^1^	NS	6	NS	NS	[12]
			2 mM	0.5 mA cm^−2^						18	NS	NS	
			4 mM	0.5 mA cm^−2^						32	NS	NS	
			6 mM	0 mA cm^−2^						20	NS	NS	
				0.25 mA cm^−2^						32	NS	NS	
				0.5 mA cm^−2^						41	NS	NS	
				0.75 mA cm^−2^						53	NS	NS	
				1 mA cm^−2^						51	NS	NS	
			8 mM	0.5 mA cm^−2^						40	NS	NS	
Paper industry wastewater	Fe/Fe	PDS	PDS/COD ratio 1.25	4.1 A	5	0.5	6.0	11700	NS	63.5	NS	NS	[15]
	Al/Al		PDS/COD ratio 0.5	4.25 A	25		7.25			72.8	NS	NS	
Greywater	Fe/Graphite	PDS	0 mM	2 mA cm^−2^	60	0.4	6.9	530	NS	50 ^1^	NS	1.81 kWh m^−3^	[13]
			8.8 mM	0 mA cm^−2^						10 ^1^	NS	NS	
				2 mA cm^−2^						68 ^1^	NS	1.49 kWh m^−3^	
Palm oil mill effluent	Al/Al	PDS	1 g L^−1 1^	20 mA cm^−2^	60	0.5	3	2420	NS	76.35	NS	NS	[33]
							5			75.79	NS	NS	
				50 mA cm^−2^			3			79.58	NS	NS	
							5			75.86	NS	NS	
			8 g L^−1 1^	20 mA cm^−2^			3			72.66	NS	NS	
							5			68.95	NS	NS	
				50 mA cm^−2^			3			74.07	NS	NS	
							5			73.24	NS	NS	
			1.784 g L^−1 1^	45 mA cm^−2^	45		4			77.7	NS	12.76 kWh m^−3^	

NS-Not specified; TSS-Total suspended solids. ^1^ Value obtained indirectly from data presented in the paper or from a figure.

**Table 3:** Summary of research results previously reported for persulfate electro-activation with metal ions addition in the treatment of complex wastewaters.

Type of Effluent	Anode/Cathode	[PDS]_added_	Iron Source	[Fe*^n^*^+^]	Applied Current	Electrolysis Time/h	Treated Volume/L	pH_0_	COD_0_/mg L^−1^	COD Removal/%	Energy Consumption/kWh kg^−1^	Reference
Landfill leachate	(Ti/IrO_2_-RuO_2_-TiO_2_)/ Ti	0 mM	FeSO_4_·7H_2_O	0 mM	13.89 mA cm^−2^	4	1	3	1900	28.1	NS	[35]
		15.6 mM		15.6 mM						55 ^1^	NS	
		31.3 mM								62 ^1^	NS	
		62.5 mM								67.7	NS	
								6		62 ^1^	NS	
								9		47.0	NS	
					0 mA cm^−2^			3		44.8	NS	
					6.94 mA cm^−2^					55 ^1^	NS	
					27.78 mA cm^−2^					62 ^1^	NS	
				7.81 mM	13.89 mA cm^−2^					55 ^1^	NS	
				31.2 mM						60 ^1^	NS	
				15.6 mM		1				62.2	5.7	
Landfill leachate concentrate	(Ti/IrO_2_)/Ti	0 mM	Fe_2_(SO_4_)_3_	0 mM	80 mA	1	0.15	7.6	1281	10.9	NS	[36]
		0 mM		15 mM	0 mA					26	NS	
		18.75 mM			80 mA					41 ^1^	NS	
		37.5 mM		0 mM	0 mA					11.9	NS	
				3.75 mM	80 mA					22 ^1^	NS	
				5 mM						30 ^1^	NS	
				7.5 mM						37 ^1^	NS	
				15 mM	0 mA					38	NS	
					40 mA					39 ^1^	NS	
					80 mA					55 ^1^	4.42	
					120 mA					50 ^1^	NS	
					160 mA					47 ^1^	NS	
		56.25 mM			80 mA					48 ^1^	NS	
		75 mM			80 mA					45 ^1^	NS	
Mixed industrial wastewater	(Ti/Pt)/Graphite felt	0 mg L^−1^	FeSO_4_·7H_2_O	20 mg L^−1^	10 V	1	1	3	1152	31 ^1^	NS	[37]
		100 mg L^−1^								31	NS	
		200 mg L^−1^		10 mg L^−1^						60	NS	
				20 mg L^−1^						60 ^1^	NS	
		300 mg L^−1^								46 ^1^	NS	
Olive mill wastewater	Pt/Graphite	200 mM	FeSO_4_·7H_2_O	20 mM	200 mA	6	0.2	5	6265	63.4	5.63	[38]
		250 mM		25 mM						71.2	4.50	
Dyeing wastewater	(Pt/Ti)/ Fe	500 mg L^−1^	FeSO_4_·7H_2_O	0 mg L^−1^	3 V	1	1	6	1024	76 ^1^	NS	[24]
				100 mg L^−1^						80	0.11	
								3		83 ^1^	NS	
								8.2		76 ^1^	NS	
								12		78 ^1^	NS	
					5 V			6		82 ^1^	1.28	
					9 V					89.4	8.45	
				250 mg L^−1^	3 V					70 ^1^	NS	

NS-Not specified. ^1^ Value obtained indirectly from data presented in the paper or from a figure.

**Table 4:** Summary of research results previously reported for persulfate electro-activation using metal-based catalysts in the treatment of complex wastewaters.

Type of Effluent	Anode/Cathode	PS Source	[PS]_added_/mM	Catalyst	[Catalyst]/g L^−1^	Applied Current	Electrolysis Time/h	Treated Volume/L	pH_0_	COD_0_/ mg L^−1^	COD Removal/%	Reference
Landfill leachate	NA	PDS	85	Fe-C	40	NA	2	0.1	7	9514	62.91	[40]
Landfill leachate	Iron filings/Hydrothermal carbonization biochar	PDS	0	Fe/C granules	0	5 V	2	0.06	7.81	1041.38	46.9	[39]
					16.(6) ^1^						50.4	
			7								47.4	
			14								55 ^1^	
			28		0						53.7	
					8.(3) ^1^						58.7	
					12.5 ^1^						66.2	
					16.(6) ^1^	0 V					4	
						1 V					36.9	
						3 V					43.1	
						5 V					72.9	
						7.5 V					73.4	
					33.(3) ^1^	5 V					73.3	
			56		16.(6) ^1^						75.6	
Landfill leachate concentrate	Stainless steel/Stainless steel	PMS	0	NrGO-MnFe_2_O_4_	0	20 mA cm^−2^	2	0.5	5.0	1250	30.12	[16]
			0		1.00	0 mA cm^−2^					9.21	
			0.5			20 mA cm^−2^					47.59	
			1.0								54 ^1^	
			1.5								65 ^1^	
			2.00		0	0 mA cm^−2^					12.17	
					0	20 mA cm^−2^					42.33	
					0.25						48 ^1^	
					0.50						60 ^1^	
					0.75						66 ^1^	
					1.00	0 mA cm^−2^					32 ^1^	
						5 mA cm^−2^					42 ^1^	
						10 mA cm^−2^					48 ^1^	
						15 mA cm^−2^					60 ^1^	
						20 mA cm^−2^					72.89	
									3.0		60 ^1^	
									4.0		65.53	
									6.0		46 ^1^	
									7.0		33.01	
									10.0		41.79	
						25 mA cm^−2^					74 ^1^	
						30 mA cm^−2^					65 ^1^	
					1.25	20 mA cm^−2^			5.0		68 ^1^	
			2.5								74.39	
Washing machine effluent	Pt/ Graphite felt	PMS	0	Fe_3_O_4_	0	30 mA cm^−2^	3	0.2	3	480	20	[26]
					0.1						47	
			0.5						5		50.9	
			1						5		64.3	
			2		0				3		32.7	
					0.025				5		44 ^1^	
					0.05				5		60 ^1^	
					0.1	0 mA cm^−2^			3		30	
						10 mA cm^−2^			5		38 ^1^	
						20 mA cm^−2^			5		52 ^1^	
						30 mA cm^−2^			3		70 ^1^	
									5		74.4	
									7		58 ^1^	
									9		57 ^1^	
						40 mA cm^−2^			5		75 ^1^	
					0.15	30 mA cm^−2^					84 ^1^	
					0.2						84 ^1^	
			3		0.1						85.4	
			4								80 ^1^	

NA-Not applied. ^1^ Value obtained indirectly from data presented in the paper or from a figure.

**Table 5:** Summary of research results previously reported for electro-persulfate processes involving co-activation by irradiation or heat in the treatment of complex wastewaters.

Type of Effluent	Anode/Cathode	PS Source	[PS]_added_	Iron Source	Irradiation	Applied Current	T/°C	Electrolysis Time/h	Treated Volume/L	pH_0_	[COD]_0_ [TOC]_0_/mg L^−1^	COD TOC Removal/%	Energy Consumption	Reference
Saline petrochemical wastewater	Fe/Graphite	PDS	0 mM	Fe anode	US: 300 W	1.2 V	21–25	2	0.3	5	COD_0_: 850	COD: 80.2	NS	[41]
								3.5				COD: 94.1	11 kWh m^−3^	
			0.25 mM					2				COD: 85 ^1^	NS	
			0.5 mM									COD: 88 ^1^	NS	
			0.75 mM									COD: 91.7	4.2 kWh m^−3^	
			1 mM									COD: 88 ^1^	NS	
Saline petrochemical wastewater	Pt / Pt	PDS	20 mM	NA	US: 100 W (35 kHz)	10 V	20	2	0.8	3	COD_0_: 750	COD: 64.8	NS	[22]
					US: 200 W (35 kHz)							COD: 67 ^1^		
					US: 300 W (35 kHz)							COD: 73.9		
					US: 0 W							COD: 69.0		
					US: 300 W (130 kHz)							COD: 82.3		
							60					COD: 91.2		
Textile effluent	Stainless Steel/Graphite	PDS	0.42 mM ^1^	NA	US: 0 W	8 V	NS	1	0.4	8.1	TOC_0_: 723	TOC: 85 ^1^	NS	[42]
					US: 44 W							TOC: 87 ^1^		
						20 V						TOC: 90		
Textile effluent	Fe/Graphite	PDS	0.5 mM	Fe anode	US: 0 W	0.5 V	NS	1.5	NS	5	COD_0_: 1250	COD: 78	NS	[43]
					US: 100 W							COD: 92 ^1^		
					US: 200 W							COD: 94 ^1^		
					US: 300 W							COD: 96		
Washing machine effluent	Pt/Graphite-felt	PMS	2 mM	Fe_3_O_4_ 0.1 g L^−1^	UV 1.02 mW cm^−2^	30 mA cm^−2^	NS	3	0.2	5	COD_0_: 480 TOC_0_: 202	COD: 99.5 TOC: 97.1	NS	[26]
Sanitary landfill leachate	Ti/IrO_2_-TaO_2_ / Ti	PDS	0 mM	FeTiO_3_ 1 g L^−1^	UV-LED 30 W	100 mA cm^−2^	25	5	0.3	8.5	TOC_0_: 5600	TOC: 12	300 kWh kg^−1^	[20]
			234 mM									TOC: 39 ^1^	87 kWh kg^−1 1^	
						50 mA cm^−2^						TOC: 23	54 kWh kg^−1 1^	
						200 mA cm^−2^						TOC: 53	234 kWh kg^−1^	
Greywater	Fe / Graphite-sheet	PDS	3 mM	Fe anode	UVC 12 W	2 mA cm^−2^	NS	0.8(3) ^1^	0.4	7	COD_0_: 530	COD: 60.3	NS	[13]
			6 mM			1 mA cm^−2^						COD: 66.9	NS	
						2 mA cm^−2^				5		COD: 71.9	NS	
										9		COD: 68.9	NS	
						3 mA cm^−2^				7		COD: 72.3	NS	
			9 mM			2 mA cm^−2^						COD: 79.6	NS	
			0 mM					1		6.9		COD: 53 ^1^	28.48 kWh m^−3^	
			8.8 mM									COD: 77	28.16 kWh m^−3^	
Sanitary landfill leachate	Ti/IrO_2_/Ti	PDS	75 mM	Fe_2_(SO_4_)_3_ 15 mM	NA	80 mA	60	2	0.15	2	COD_0_: 1281	COD: 50 ^1^	NS	[23]
							70					COD: 58 ^1^	NS	
							80					COD: 87	91.9 kWh kg^−1^	
							90					COD: 87 ^1^	NS	

NA-Not applied; NS-Not specified. ^1^ Value obtained indirectly from data presented in the paper or from a figure.

**Table 6:** Strengths and weaknesses of the hybrid persulfate electro-activation process.

Activation Method Besides Electrochemical	Strengths	Weaknesses
Metal sacrificial anodes	Low-cost and high efficiency Metal addition may be controlled by current PS/Metal/COD ratio may be easily controlled	Sludge formation with metal content if high currents are utilized Better results at low pH, which leads to acidic metal dissolution and sulfate radical scavenging
Metal catalysts	Low-cost and high efficiency Formation of metal hydroxide sludge hindered Possibility of catalysts recycling Possible recovery by external magnets if the material has magnetic properties Possibility of using natural catalysts, such as pyrite and chalcopyrite	Metal ion leaching Poor stability and reusability Limitation in catalysts recovery
UV or visible radiation	Clean and easy to handle Environmentally friendly if solar energy can be utilized	Inadequate for dark wastewaters or effluents containing suspended solids
Ultrasound	Microstreaming and cavitation bubble generation, eliminating mass transfer resistance Increased PDS decomposition and hydroxyl and sulfate radical production	Increase in the treatment cost
Heat	Increase in the degradation reactions rate	Energy spent in heating the wastewater Possibility of increasing side unwanted reactions

## Data Availability

No new data were created or analyzed in this study. Data sharing is not applicable to this article.

## References

[1] Shrivastava P., Naoghare P.K., Gandhi D., Devi S.S., Krishnamurthi K., Bafana A., Kashyap S.M., Chakrabarti T. (2017). Application of cell-based assays for toxicity characterization of complex wastewater matrices: Possible applications in wastewater recycle and reuse. Ecotoxicol. Environ. Saf..

[2] Brillas E., Martínez-Huitle C.A. (2015). Decontamination of wastewaters containing synthetic organic dyes by electrochemical methods. An updated review. Appl. Catal. B.

[3] Giannakis S., Lin K.-Y.A., Ghanbari F. (2021). A review of the recent advances on the treatment of industrial wastewaters by Sulfate Radical-based Advanced Oxidation Processes (SR-AOPs). Chem. Eng. J..

[4] Wang J., Wang S. (2018). Activation of persulfate (PS) and peroxymonosulfate (PMS) and application for the degradation of emerging contaminants. Chem. Eng. J..

[5] Zhi D., Lin Y., Jiang L., Zhou Y., Huang A., Yang J., Luo L. (2020). Remediation of persistent organic pollutants in aqueous systems by electrochemical activation of persulfates: A review. J. Environ. Manag..

[6] Yang S.-Q., Cui Y.-H., Liu Y.-Y., Liu Z.-Q., Li X.-Y. (2018). Electrochemical generation of persulfate and its performance on 4-bromophenol treatment. Sep. Purif. Technol..

[7] Karim A.V., Jiao Y., Zhou M., Nidheesh P.V. (2021). Iron-based persulfate activation process for environmental decontamination in water and soil. Chemosphere.

[8] Ganiyu S.O., El-Din M.G. (2020). Insight into in-situ radical and non-radical oxidative degradation of organic compounds in complex real matrix during electrooxidation with boron doped diamond electrode: A case study of oil sands process water treatment. Appl. Catal. B.

[9] Matzek L.W., Tipton M.J., Farmer A.T., Steen A.D., Carter K.E. (2018). Understanding electrochemically activated persulfate and its application to ciprofloxacin abatement. Environ. Sci. Technol..

[10] Matzek L.W., Carter K.E. (2016). Activated persulfate for organic chemical degradation: A review. Chemosphere.

[11] Guvenc S., Varank G., Demir A., Guvenk E. (2020). Energy consumption and efficiency improvement of electro-activated persulfate processes: Optimization by CCD for TOC Removal from leachate concentrate. Sigma J. Eng. Nat. Sci..

[12] Jaafarzadeh N., Ghanbari F., Alvandi M. (2017). Integration of coagulation and electro-activated HSO_5−_to treat pulp and paper wastewater. Sustain. Environ. Res..

[13] Ahmadi M., Ghanbari F. (2016). Optimizing COD removal from greywater by photoelectro-persulfate process using Box-Behnken Design: Assessment of effluent quality and electrical energy consumption. Environ. Sci. Pollut. Res..

[14] Yuan S., Liao P., Alshawabkeh A.N. (2014). Electrolytic manipulation of persulfate reactivity by iron electrodes for trichloroethylene degradation in groundwater. Environ. Sci. Technol..

[15] Varank G., Guvenc S., Demir A., Kavan N., Donmez N., Onen Z. (2020). Modeling and optimizing electro-persulfate processes using Fe and Al electrodes for paper industry wastewater treatment. Water Sci. Tech..

[16] Wang W., Chen M., Wang D., Yan M., Liu Z. (2021). Different activation methods in sulfate radical-based oxidation for organic pollutants degradation: Catalytic mechanism and toxicity assessment of degradation intermediates. Sci. Total Environ..

[17] Liu H., Bruton T.A., Doyle F.M., Sedlak D.L. (2014). In situ chemical oxidation of contaminated groundwater by persulfate: Decomposition by Fe(III)- and Mn(IV)-containing oxides and aquifer materials. Environ. Sci. Technol..

[18] Oh W.D. (2016). Activation of Peroxymonosulfate by Heterogeneous Catalysts for the Removal of Organic Pollutants in Water. Ph.D. Thesis.

[19] Oh S.-Y., Kang S.-G., Chiu P.C. (2010). Degradation of 2,4-dinitrotoluene by persulfate activated with zero-valent iron. Sci. Total Environ..

[20] Silveira J.E., Zazo J.A., Pliego G., Casas J.A. (2018). Landfill leachate treatment by sequential combination of activated persulfate and Fenton oxidation. Waste Manag..

[21] Darsinou B., Frontistis Z., Antonopoulou M., Konstantinou I., Mantzavinos D. (2015). Sono-activated persulfate oxidation of bisphenol A: Kinetics, pathways and the controversial role of temperature. Chem. Eng. J..

[22] Yousefi N., Pourfadakari S., Esmaeili S., Babaei A.A. (2019). Mineralization of high saline petrochemical wastewater using Sonoelectro-activated persulfate: Degradation mechanisms and reaction kinetics. Microchem. J..

[23] Xue W., Cui Y., Liu Z., Yang S., Li J., Guo X. (2020). Treatment of landfill leachate nanofiltration concentrate after ultrafiltration by electrochemically assisted heat activation of peroxydisulfate. Sep. Purif. Technol..

[24] Chanikya P., Nidheesh P.V., Babu D.S., Gopinath A., Kumar M.S. (2021). Treatment of dyeing wastewater by combined sulfate radical based electrochemical advanced oxidation and electrocoagulation processes. Sep. Purif. Technol..

[25] Chen W.-S., Jhou Y.-C., Huang C.-P. (2014). Mineralization of dinitrotoluenes in industrial wastewater by electro-activated persulfate oxidation. Chem. Eng. J..

[26] Ghanbari F., Martínez-Huitle C.A. (2019). Electrochemical advanced oxidation processes coupled with peroxymonosulfate for the treatment of real washing machine effluent: A comparative study. J. Electroanal. Chem..

[27] Yang W., Liu G., Chen Y., Miao D., Wei Q., Li H., Ma L., Zhou K., Liu L., Yu Z. (2020). Persulfate enhanced electrochemical oxidation of highly toxic cyanide-containing organic wastewater using boron-doped diamond anode. Chemosphere.

[28] Onn S., Bashir M., Sethupathi S., Amr S., Nguyen T. (2020). Colour and COD removal from mature landfill leachate using electro-persulphate oxidation process. Mater. Today Proc..

[29] Varank G., Guvenc S., Dincer K., Demir A. (2020). Concentrated leachate treatment by electro-Fenton and electro-persulfate processes using Central Composite Design. Int. J. Environ. Res..

[30] Varank G., Guvenc S., Demir A. (2020). Electro-activated peroxymonosulfate and peroxydisulfate oxidation of leachate nanofiltration concentrate: Multiple-response optimization. Int. J. Environ. Sci. Tech..

[31] Guvenc S., Varank G. (2020). Box-Behnken Design optimization of electro-Fenton/-persulfate processes following the acidification for TSS removal from biodiesel wastewater. Sigma J. Eng. Nat. Sci..

[32] Guvenc S., Varank G., Cebi A., Ozkaya B. (2021). Electro-activated persulfate oxidation of biodiesel wastewater following acidification phase: Optimization of process parameters using Box–Behnken Design. Water Air Soil Pollut..

[33] Bashir M., Wei C., Aun N., Amr S. (2017). Electro persulphate oxidation for polishing of biologically treated palm oil mill effluent (POME). J. Environ. Manag..

[34] Durna E., Genç N. (2021). Application of a multiple criteria analysis for the selection of appropriate radical based processes in treatment of car wash wastewater. Environ. Eng. Res..

[35] Zhang H., Wang Z., Liu C., Guo Y., Shan N., Meng C., Sun L. (2014). Removal of COD from landfill leachate by an electro/Fe^2+^/peroxydisulfate process. Chem. Eng. J..

[36] Cui Y.H., Xue W.J., Yang S.Q., Tu J.L., Guo X.L., Liu Z.Q. (2018). Electrochemical/peroxydisulfate/Fe^3+^ treatment of landfill leachate nanofiltration concentrate after ultrafiltration. Chem. Eng. J..

[37] Popat A., Nidheesh P.V., Singh T.A., Kumar M.S. (2019). Mixed industrial wastewater treatment by combined electrochemical advanced oxidation and biological processes. Chemosphere.

[38] Görmez F., Görmez Ö., Yabalak E., Gözmen B. (2020). Application of the central composite design to mineralization of olive mill wastewater by the electro/FeII/persulfate oxidation method. SN Appl. Sci..

[39] Yu D., Cui J., Li X., Zhang H., Pei Y. (2020). Electrochemical treatment of organic pollutants in landfill leachate using a three-dimensional electrode system. Chemosphere.

[40] Zhang W., Li X., Yang Q., Wang D., Wu Y., Zhu X., Wei J., Liu Y., Hou L., Chen C. (2019). Pretreatment of landfill leachate in near-neutral pH condition by persulfate activated Fe-C micro-electrolysis system. Chemosphere.

[41] Ahmadi M., Haghighifard N.J., Soltani R.D.C., Tobeishi M., Jorfi S. (2019). Treatment of a saline petrochemical wastewater containing recalcitrant organics using electro-Fenton process: Persulfate and ultrasonic intensification. Desalin. Water Treat..

[42] Johin J., Nidheesh P.V., Sivasankar T. (2019). Sono-electro-chemical treatment of Reactive Black 5 dye and real textile effluent using MnSO_4_/Na_2_S_2_O_8_ electrolytes. Arab. J. Sci. Eng..

[43] Jorfi S., Ghaedrahmat Z. (2020). Evaluating the efficiency of advanced oxidation processes for textile wastewater treatment: Electro-kinetic, sonochemical and persulfate. Environ. Prog. Sustain. Energy.

[44] Trellu C., Chaplin B.P., Coetsier C., Esmilaire R., Cerneaux S., Causserand C., Cretin M. (2018). Electro-oxidation of organic pollutants by reactive electrochemical membranes. Chemosphere.

[45] Chaplin B.P. (2019). The prospect of electrochemical technologies advancing worldwide water treatment. Acc. Chem. Res..

[46] Chuah C.Y., Lee J., Bae T.-H. (2020). Graphene-based membranes for H_2_ separation: Recent progress and future perspective. Membranes.

[47] Wang J., Wang S. (2021). Toxicity changes of wastewater during various advanced oxidation processes treatment: An overview. J. Clean Prod..

